# Upscaling Chamber-measured Greenhouse Gas Emissions in Rewetted Peat Extraction Sites Using Drone and Satellite Imagery

**DOI:** 10.1007/s00267-026-02582-2

**Published:** 2026-08-18

**Authors:** Aleksi Isoaho, Milla Niiranen, Eveliina Väyrynen, Aleksi Räsänen, Maarit Liimatainen

**Affiliations:** 1 Natural Resources Institute Finland (Luke), Oulu, Finland; 2 Natural Resources Institute Finland (Luke), Ruukki, Finland; 3 Geography Research Unit, Faculty of Science, University of Oulu, Oulu, Finland; 4 Water, Energy and Environmental Engineering Research Unit, Faculty of Technology, University of Oulu, Oulu, Finland

**Keywords:** Remote sensing, Peatland, Wetland, UAV, Sentinel-2, Greenhouse gas

## Abstract

Greenhouse gas (GHG) emissions originating from rewetted peat extraction sites are largely unknown. While the emissions can be quantified with chamber measurements, spatial upscaling is needed to quantify the emissions over the whole rewetted areas. Therefore, we developed a method to upscale chamber-measured GHG emissions with drone and satellite imagery. We measured GHG fluxes in five different surface types within three rewetted peatlands in northern Finland across two growing seasons. We spatially classified surface types within drone mapped areas with random forest classifier and further upscaled the surface type %-covers outside the drone areas with multitemporal Sentinel-2 imagery using extreme gradient boosting regression. With predicted surface type %-covers, we calculated total and area-normalised GHG flux sums for each measurement moment. The drone-based surface type classifiers performed well (class specific F-scores 0.28–0.97, overall accuracies 0.76–0.85) as did the satellite-based surface type %-cover models (R^2^ = 0.38–0.99). Predicted surface type covers appeared realistic and followed topographical gradient observed from the high-resolution classification. Upscaled and area-normalised GHG fluxes ranged from 0.24 to 5.91 g CO2-C m^–2^ d^–1^, 0.01 to 0.34 g CH4-C m^–2^ d^–1^ and -98 to 61 μg N2O-N m^–2^ d^–1^ depending on site and time. High water and reed %-cover reduced overall emissions due to higher emission factors in drier surface types but due to lack of gross primary production estimations, no conclusion could be drawn about net ecosystem carbon balances. Our results indicate that combination of field measurements, drone flights, and satellite imagery can be used to upscale surface area estimates and GHG fluxes.

## Introduction

Peatlands are critical ecosystems providing carbon storage (Joosten et al. 2016), unique habitats for species (Chapman et al. 2003), and water regulation (Ramchunder et al. 2012). Human-induced drainage has largely impacted peatlands, with 11% of them being strongly altered (Leifeld and Menichetti 2018). Artificial drainage lowers the water table depth, which exposes peat to aerobic decomposition (Whittington and Price 2006; Itoh et al. 2017), causing greenhouse gas (GHG) emissions that accelerate climate change (Leifeld and Menichetti 2018; Wilkinson et al. 2023).

Peat extraction for horticultural and energy purposes has contributed to around 10% of the loss of peatlands outside the tropical zone (Joosten and Clarke 2002). In Finland, former and current peat extraction areas cover up to around 135,000 ha (Syke 2023) while recently peat extraction has been significantly reduced and a large number of sites have been shut down. After the peat extraction has ceased, several after-use practices can occur in the peat extraction site (Räsänen et al. 2023). One of the most popular after-uses is to rewet the site by creating shallow water bodies (e.g. Kozlov et al. 2016; Lundin et al. 2017; Reddin et al. 2025). The water bodies enable plant colonization (Kozlov et al. 2016) and increase anoxic conditions that reduce carbon dioxide (CO_2_) and nitrous oxide (N_2_O) emissions but promote methane (CH_4_) emissions (Minke et al. 2016; Jordan et al. 2020). Rewetted peat extraction sites typically consist of different microsites and larger landscape features such as open water areas and reed islands. Consequently, there can be large spatial differences in the GHG fluxes—part of the site can act as a carbon sink while other parts of the site can be carbon sources depending on local vegetation, soil, and hydrological characteristics (Minke et al. 2016; Jordan et al. 2020; Ge et al. 2024). As former peat extraction sites are often relatively large, laborious field-based monitoring across the whole site is not usually feasible. Therefore, more localised but representative field measurements need to be combined with spatial upscaling.

Remote sensing, particularly drone and satellite observations, has been used to spatially monitor and upscale different field-measured ecosystem functions and characteristics such as land cover, vegetation patterns, water table depth, and GHG fluxes (Steenvoorden et al. 2023; Isoaho et al. 2024b, a; Villoslada et al. 2024). Satellite imagery has been widely used to produce large-area estimates of GHG fluxes by linking GHG measurements to categorical land cover or vegetation classifications or continuous remotely sensed variables through regression methods (Davidson et al. 2017; Räsänen et al. 2021; Ludwig et al. 2024). Satellite imagery typically has a relatively coarse spatial resolution (≥ 10 m pixel size in freely accessible datasets) but seamless global availability with frequent revisit time. Instead, drone datasets enable ultra-high spatial resolution (cm-level pixel size) characterisation of peatland GHG fluxes, yet with a limited spatial and temporal extent (Lehmann et al. 2016; Kelly et al. 2021; Sjögersten et al. 2023). It has been shown that in highly heterogeneous landscapes such as peatlands (Räsänen and Virtanen 2019), GHG upscaling using coarse-resolution data can be biased due to inaccurate area estimates (Hashemi et al. 2025; Ivanova et al. 2026). Furthermore, the chamber measurements typically cover areas notably smaller than the pixel size of satellite imagery, causing a spatial mismatch. Therefore, there is a methodological need to combine coarse-resolution satellite products with high-resolution drone imagery to produce GHG flux estimates over large spatial extents.

One possible solution for the combination is to (1) link field-measured GHG fluxes to a drone-based vegetation, land, or surface cover classification within a small area and (2) upscale the classification and GHG flux estimates to a larger area covered by satellites through surface type-specific regression models. In this approach, GHG fluxes would thus be linked to percentage-cover estimates of surface type classes within satellite pixels instead of one categorical class for each pixel. A similar approach has been used for vegetation, land cover, and wetness characteristics (Riihimäki et al. 2019; Bergamo et al. 2023; Keränen et al. 2024; Villoslada et al. 2024; Putkiranta et al. 2025; Klehr et al. 2025) but not yet for GHG fluxes. Drone data is needed as a mediator between satellite pixels and field plots since the field measurements usually cover only a small proportion of a satellite pixel; for instance, chamber-based GHG flux measurements cover typically < 1 m^2^ and satellite pixels ≥100 m^2^. Therefore, satellite pixel %-cover-based upscaling could provide more accurate estimations compared to satellite pixel class-based approach.

To develop and test GHG flux upscaling for rewetted peat extraction sites, we link chamber-measured ecosystem respiration (CO_2_ emissions from autotrophic and heterotrophic respiration, hereafter *R*_eco_), CH_4_, and N_2_O fluxes from three sites in northern Finland to drone and satellite data. Our specific aims are to (1) upscale surface types in high spatial resolution using multispectral drone imagery with a limited extent, (2) upscale surface type areas across the entire site using multitemporal satellite imagery, and (3) calculate landscape-scale GHG emissions based on the areal extent of surface types and chamber measurements.

## Materials and Methods

### Study Sites and Field Data

**Fig. 1:**
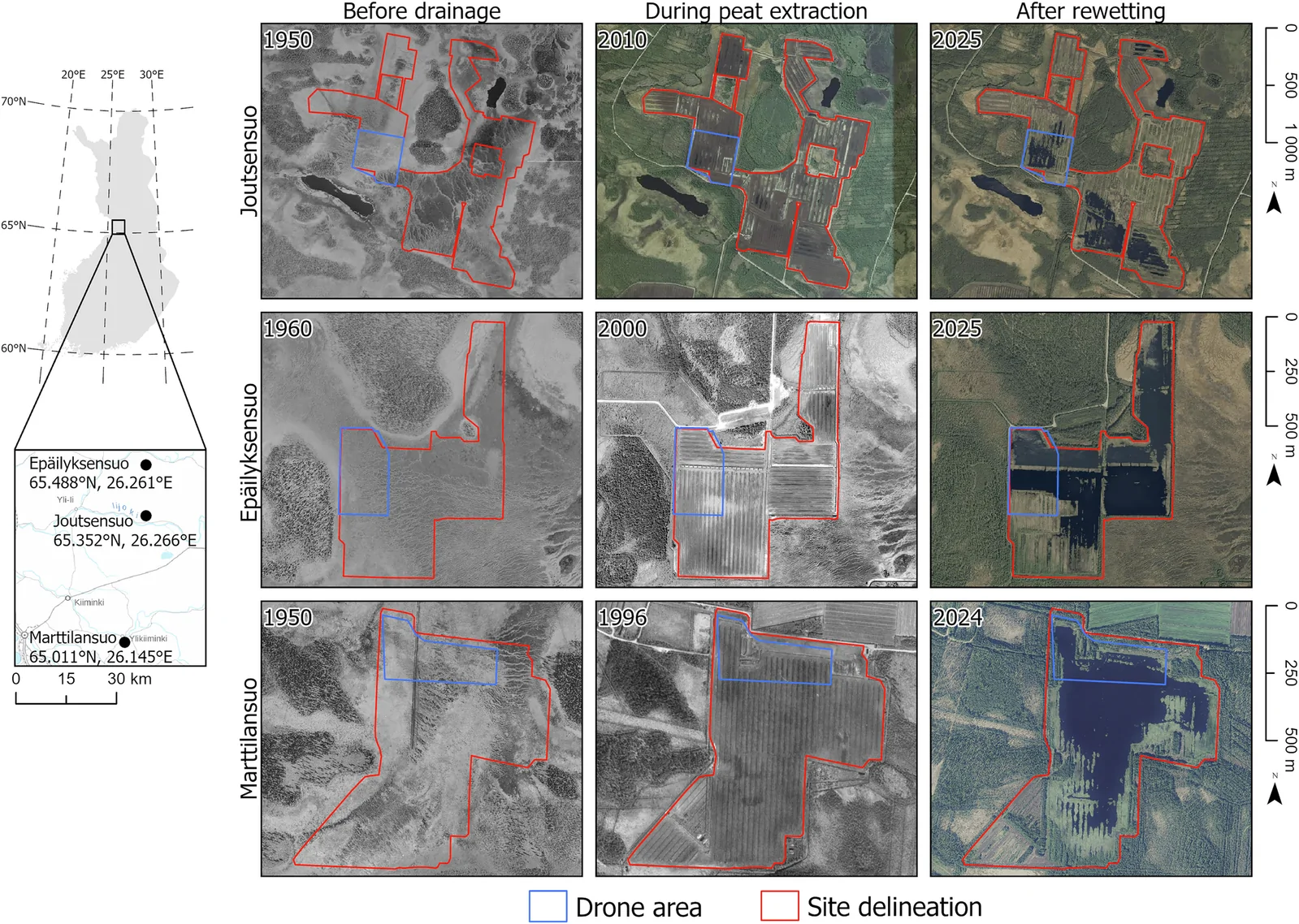
Study sites before drainage, during peat extraction, and after rewetting. Numbers within aerial images indicate their acquisition years. Aerial images and background map are open data from National Land Survey of Finland

We studied three rewetted peat extraction sites in northern Finland (Fig. 1). In their natural state, the sites were minerotophic fens and part of larger aapa mire complexes (Sallinen et al. 2019), but they were drained for peat extraction in the 1970s–1990s. After the peat extraction ceased, sites were ash-fertilized and abandoned for one to three years after which they were partly rewetted in 2019 (Joutsensuo), 2013 (Epäilyksensuo), and 2009 (Marttilansuo). The most recently rewetted Joutsensuo has the lowest proportion of open water and has remained relatively open with low tree cover. In Epäilyksensuo and Marttilansuo, open water covers around half of the sites while the drier parts of the sites are partly covered by trees and tall shrubs.—

From the study sites, we identified six clearly distinct surface types (Table 1) that differed from each other based on plant functional type covers (Fig. S1). We monitored these surface types with opaque closed chambers between May and September in 2024 and 2025, and measured *R*_eco_, CH_4_, and N_2_O exchange once a month (see exact measurement dates in supplementary material Table S1). Each site had three measurement plots for each surface type. However, the surface type “bare peat” was only monitored in Joutsensuo and the type “tall shrubs” was not monitored at all as it was not possible with chambers due to tall (>1 m) vegetation. Collars with water-filled grooves were permanently inserted 20–40 cm into the soil to ensure an airtight seal, and fluxes were measured using an opaque chamber (60 × 60 x 20–40 cm) connected to portable LI-COR LI-7810 and LI-7820 gas analysers. Measurements from open water and reed were conducted with a floating chamber (⌀30 × 40 cm). The chambers had a vent for pressure stabilization. Air temperature inside the chamber was recorded at the beginning and end of each measurement. Each measurement lasted three minutes, from which we calculated fluxes (g m^–2^ d^–1^) based on ideal gas law and linear regression. All concentration time series were visually inspected, and data affected by chamber leakage or ebullition events were excluded. As one dry vegetation plot from Joutsensuo showed suspiciously high CH_4_ fluxes across the two growing seasons, it was excluded from further analysis.

**Table 1:** Surface type classes and their descriptions within the sites

Class	Description
Bare peat	Bare peat without vegetation. Greenhouse gas (GHG) fluxes were measured only in Joutsensuo due to low cover of bare peat in the two other sites. Joutsensuo measurements were utilised as a reference for Marttilansuo. In Epäilyksensuo, the class was not utilised.
Tall shrubs	Trees and tall shrubs, merged into dry vegetation for GHG assessments because GHG fluxes of tall shrubs could not be measured with chambers.
Dry vegetation	Grasses and mineral forest-type vegetation with some mire species; water table depth below the surface.
Wet vegetation	*Carex*-dominated vegetation with some *Sphagnum* and other wet mire species (such as *Eriophorum vaginatum*); water table depth very close or above the surface.
Reed	Usually at the very end of littoral zone; aquatic reed grasses, aquatic *Carex*, other aquatic plants; water table depth above the surface.
Water	Full water %-cover, no vegetation above the water surface.

Plant functional type covers for each class within each site can be seen from Supplementary material Fig. S1.

We assessed whether GHG fluxes differed between each surface type pairs by conducting pairwise Mann–Whitney U tests using all measurements. Additionally, we conducted Kruskal-Wallis test over all surface types to assess whether there were differences between the types in total. We conducted statistical tests for each site separately.

To generate GHG coefficients, i.e. gas specific emission factors for each surface type per measurement moment, we calculated average GHG coefficients over measured fluxes per surface type per site. As we did not measure tall shrubs, we used dry vegetation coefficients for *R*_eco_ and N_2_O fluxes as reference because they were in line with existing literature on measurements from afforested peat extraction sites (Mäkiranta et al. 2007; Bravo et al. 2018; Buzacott et al. 2025). However, for CH_4_, we used a constant value (0.002928 g CH_4_-C m^–2^ d^–1^) from Buzacott et al. (2025) as our measured dry vegetation CH_4_ coefficients were notably higher than the measured CH_4_ emissions from afforested peat extraction sites in earlier studies.

### Remote Sensing Data

We used ultra-high spatial resolution drone and medium spatial resolution satellite multispectral imagery in our upscaling analysis (see image capture dates in Table S2). We conducted drone flights in July and August 2025 using DJI Matrice 350 RTK drone and AgEagle MicaSense Altum-PT sensor on consistent overcast days which minimized the effect of shadows within the study sites. The flights were conducted with the speed of 9 m/s, side and front overlaps of 75%, and an altitude of 118 m from the take-off point. The captured images were converted into orthomosaics with spatial resolution of ~5 cm using structure-from-motion procedure in Agisoft Metashape (version 2.0.3) and radiometrically calibrated using MicaSense reflectance panel and irradiance sensor.

We used Sentinel-2 Level-2A reflectance product as our medium resolution multispectral imagery. We manually selected four cloud-free images from different phenological moments within the growing season 2025 for each site separately (Table S2) and projected the images to Finnish coordinate system (EPSG: 3067) with a spatial resolution of 10 m using nearest neighbour interpolation.

From all multispectral data, we calculated several spectral vegetation and moisture indices (Table 2). Additionally, we used canopy height model (CHM; spatial resolution of 1 m) calculated by Finnish Forest Centre from LiDAR data captured by National Land Survey of Finland. LiDAR data were captured in summers 2022 for Joutsensuo and Epäilyksensuo, and in 2024 for Marttilansuo.

**Table 2:** Used remote sensing variables and their abbreviations, equations, references, and data sources

Predictor	Abbreviation	Equation	Reference	Source
Blue reflectance	Blue			Drone, Sentinel-2
Green reflectance	Green			Drone, Sentinel-2
Red reflectance	Red			Drone, Sentinel-2
Red Edge reflectance	RE			Drone
Red Edge reflectance 1	RE1			Sentinel-2
Red Edge reflectance 2	RE2			Sentinel-2
Red Edge reflectance 3	RE3			Sentinel-2
Red Edge reflectance 4	RE4			Sentinel-2
Near-infrared reflectance	NIR			Drone, Sentinel-2
Shortwave infrared reflectance 1	SWIR1			Sentinel-2
Shortwave infrared reflectance 2	SWIR2			Sentinel-2
Normalised difference vegetation index	NDVI	$\frac{{NIR}-{Red}}{{NIR}+{Red}}$	Tucker 1979	Drone, Sentinel-2
Enhanced vegetation index	EVI	$2.5* \frac{{NIR}-{Red}}{{NIR}+6\;*\; {Red}-7.5\;*\; {Blue}+1}$	Liu and Huete 1995	Drone, Sentinel-2
Soil adjusted vegetation index	SAVI	$1.5* \frac{{NIR}-{Red}}{{NIR}+{Red}+0.5}$	Huete 1988	Drone, Sentinel-2
Normalised difference red edge index	NDRE	$\frac{{NIR}-{RE}}{{NIR}+{RE}}$	Barnes et al. 2000	Drone, Sentinel-2
Normalised difference water index	NDWI	$\frac{{Green}-{NIR}}{{Green}+{NIR}}$	McFeeters 1996	Drone, Sentinel-2
Shortwave infrared transformed reflectance	STR	$\frac{{(1-{SWIR}1)}^{2}}{2* {SWIR}1}$	Sadeghi et al. 2015	Sentinel-2
Normalised difference moisture index	NDMI	$\frac{{NIR}-{SWIR}1}{{NIR}+{SWIR}1}$	Gao 1996	Sentinel-2
Normalised difference moisture index 2	NDMI2	$\frac{{NIR}-{SWIR}2}{{NIR}+{SWIR}2}$	Gao 1996	Sentinel-2
Moisture stress index	MSI	$\frac{{SWIR}1}{{NIR}}$	Vogelmann and Rock 1986	Sentinel-2
Canopy height model	CHM			LiDAR

### High-resolution Surface Type Classifications

To build drone-based surface type classifications for each site, we built training and validation data with stratified random sampling. We first average-resampled drone bands and indices into 50 cm spatial resolution after which we conducted unsupervised classification to 15 different clusters with Iso Clustering (Tou and Gonzales 1974). For each cluster, we randomly sampled 20 points for which we assigned surface type class based on visual interpretation of original-resolution drone imagery and experience accumulated during field visits. In Marttilansuo, our stratified random sampling included only four points for the class bare peat due to its low %-cover in the drone-covered area; therefore, we manually added 20 bare peat points to improve the classification as underrepresent classes are often poorly classified (Khan et al. 2024). For each point, we generated 25 cm radius buffers for which we extracted means and standard deviations of the original-resolution drone predictors (Table 2). For CHM, we only calculated mean due to its lower 1 m spatial resolution. Additionally, we calculated standard deviations of drone predictors and average CHM for the 50 cm resolution grid used for sampling to produce spatial predictions.

We classified surface types with the random forest machine learning algorithm (Breiman 2001) due to its ability to handle predictor multicollinearity and non-linear relationships. To address spatial autocorrelation, we used spatial clustering (Brenning 2012; Mahoney et al. 2023) to place sample into 10 spatial folds which were used for tuning and validating the classifiers. We set number of trees to 500 as performance usually stabilises after 100 trees (Rodriguez-Galiano et al. 2012; Probst et al. 2019) and we grid searched optimal mtry using *ranger* and *mlr3* packages (Wright and Ziegler 2017; Lang et al. 2019) in R (version 4.5.2). We assessed the classification performance by calculating class-specific precision, recall, and F-score and the overall accuracy of the entire multi-class classification. With the constructed classifiers, we predicted classes across the 50 cm resolution grid to produce spatial layers with distinct surface classes

### Upscaling surface type %-covers and emissions outside drone areas

**Fig. 2:**
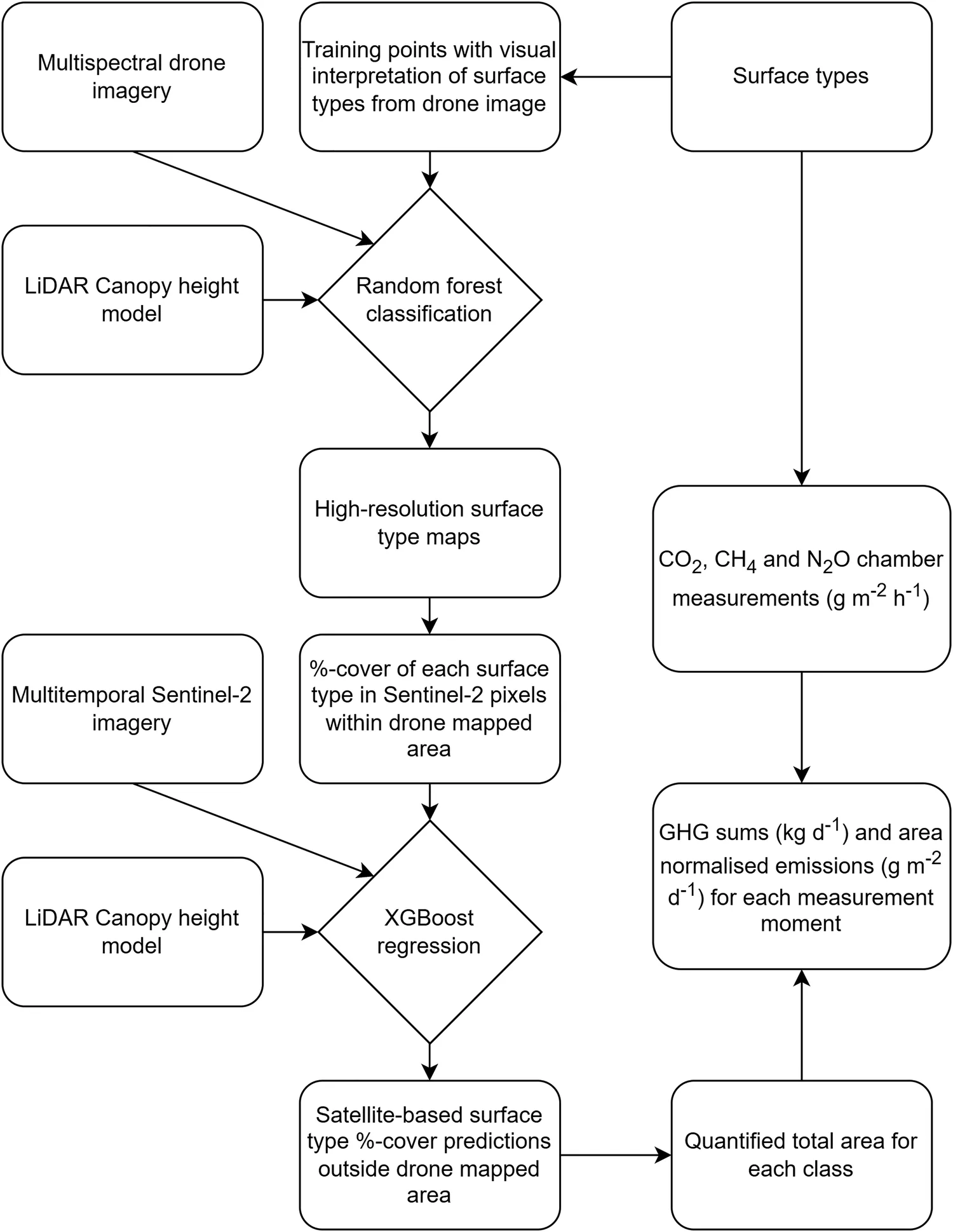
Simplified methodological flow chart of the study. GHG refers to greenhouse gas

We upscaled %-cover of drone-derived surface types to Sentinel-2 pixels (Fig. 2). To generate training and validation data, we calculated proportional cover of the drone-classified surface types for each Sentinel-2 pixel within the drone areas. In Marttilansuo, we lacked large bare peat areas within the drone area; therefore, we visually digitalised extra polygons with peat cover from aerial image of National Land Survey of Finland captured in summer of 2024. With the extra bare peat polygons, we calculated proportional covers of the bare peat within the Sentinel-2 pixels (n = 38) which intersected the polygons.

We used extreme gradient boosting (XGBoost; Chen and Guestrin 2016) regression for upscaling the drone-estimated surface type %-covers with predictors being multitemporal Sentinel-2 variables and 10 m average-resampled CHM. XGBoost is able to predict extreme values relatively well (Meinshausen 2006; Velthoen et al. 2023) which was essential in our case as our response variables (surface type %-covers) had a fixed range of 0 to 100 with a relatively large number of 0 s (e.g. other classes than water in water-covered areas) and 100 s (water in water-covered areas). We conducted the modelling separately for each site and fine-tuned the hyperparameters for each model using randomised search with spatial tenfold cross validation generated with spatial clustering and 100 optimisation rounds (see Table S3 for the tuned values) with *mlr3* package. Similarly, we validated the models with spatial tenfold cross validation. We calculated explained variance (R^2^; 1 – mean square error / variance of response variable), root mean square error (RMSE) and normalised root mean square error (nRMSE, RSME divided by the range of response variable) to assess the model performance.

With the constructed models, we predicted surface type %-covers over the entire study sites outside the drone-covered areas. From the predictions, we normalised each pixel %-cover sum to be 100. In most of the cases, the normalisation adjusted the predicted values only a few %-points. From the normalised predictions, we summarised the areas for each surface type. Using the surface type area sums, we calculated the momentary GHG flux sums based on the field-measured and surface type-averaged GHG coefficients. Additionally, we calculated area normalized means for GHG fluxes for each measurement moment by dividing total GHG flux sums with the total area of sites.

## Results

### High-resolution Surface Type Classifications

**Fig. 3:**
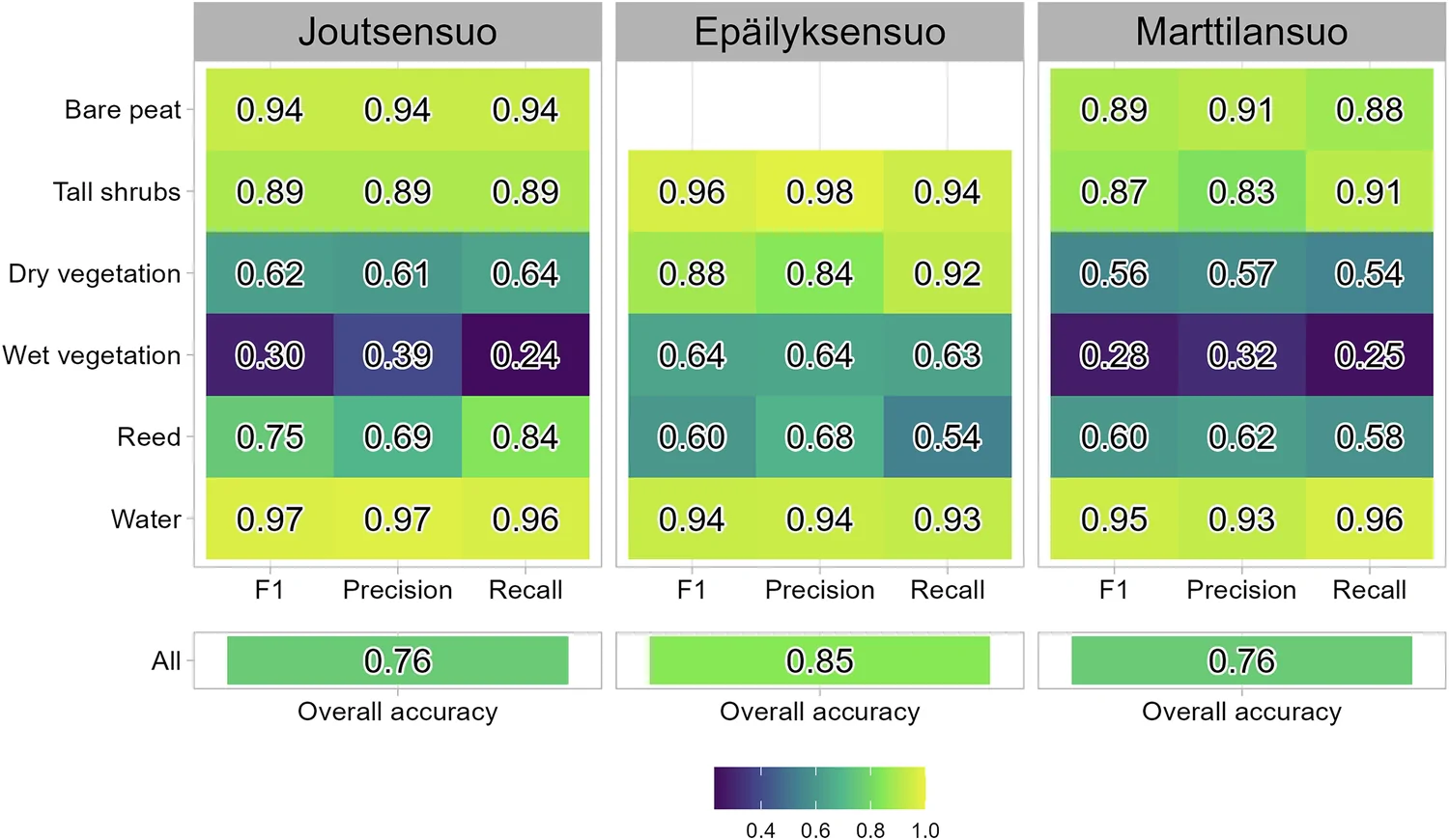
Class-specific and overall performance metrics of the drone and canopy height model-based high spatial resolution surface type classifications for each site. F1 refers to F-score

**Fig. 4:**
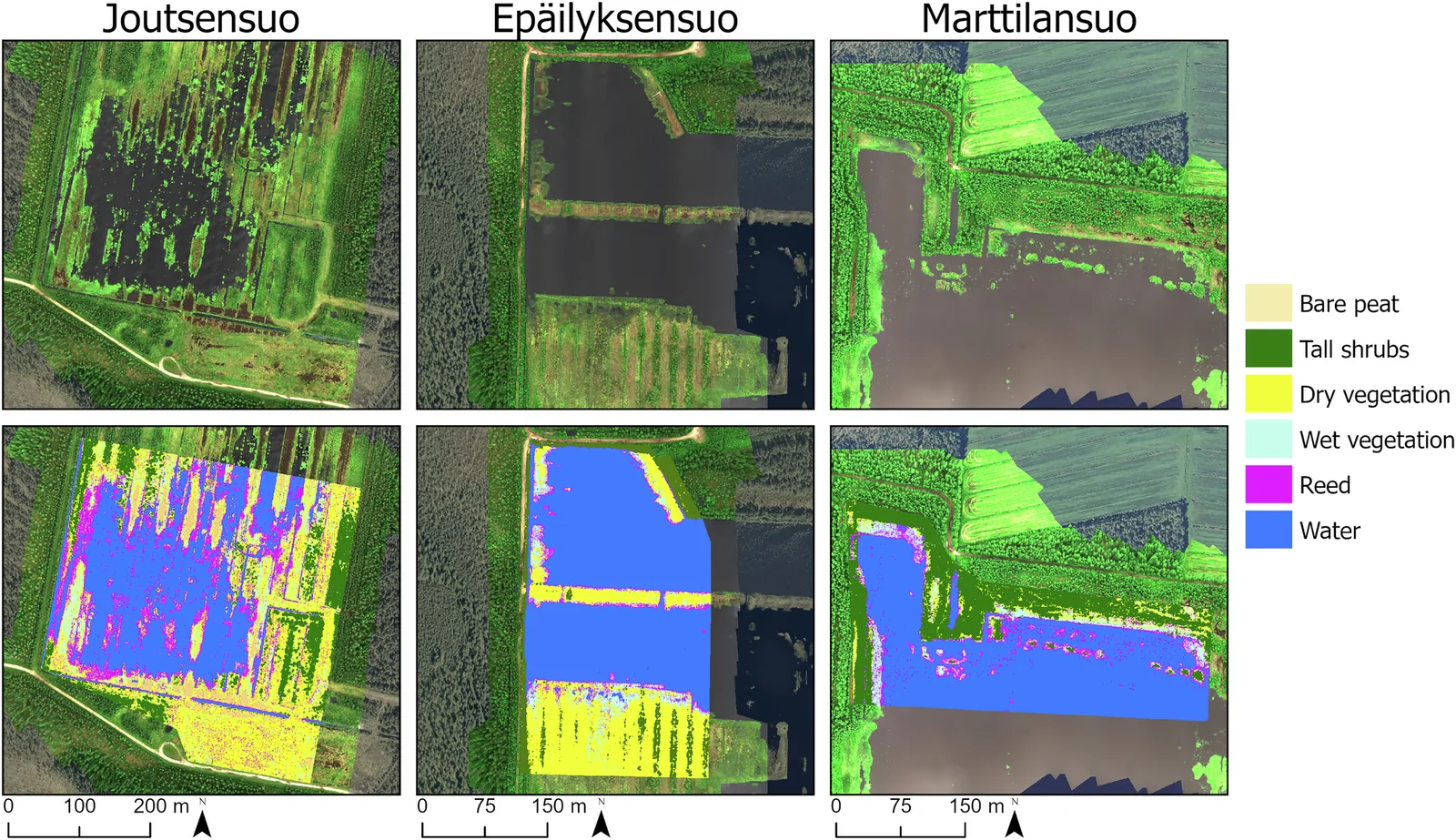
Drone-derived high resolution surface type maps (bottom panel) and RGB-presentations from constructed orthomosaics (top panel). Background aerial images are open data from National Land Survey of Finland

Drone and CHM-based surface type classification performed mostly well (class-specific F-scores 0.28–0.97, overall accuracies 0.76–0.85; Fig. 3). There was some variation in the accuracy between the types, with water (F-scores = 0.94–0.97), tall shrubs (0.87–0.96), and bare peat (0.89–0.94) being the most accurately classified, while accuracies were lower for reed (0.60–0.75), wet vegetation (0.30–0.64) and dry vegetation (0.56–0.88). Generally, the classifiers performed the best in Epäilyksensuo. Classification maps showed spatial variation and relatively high spatial heterogeneity of classes (Fig. 4). There were some salt-and-pepper-like misclassifications especially in dry vegetation areas in Joutsensuo but in general the patterns looked realistic.

### Medium-resolution Surface Type %-cover Regression Performances

**Fig. 5:**
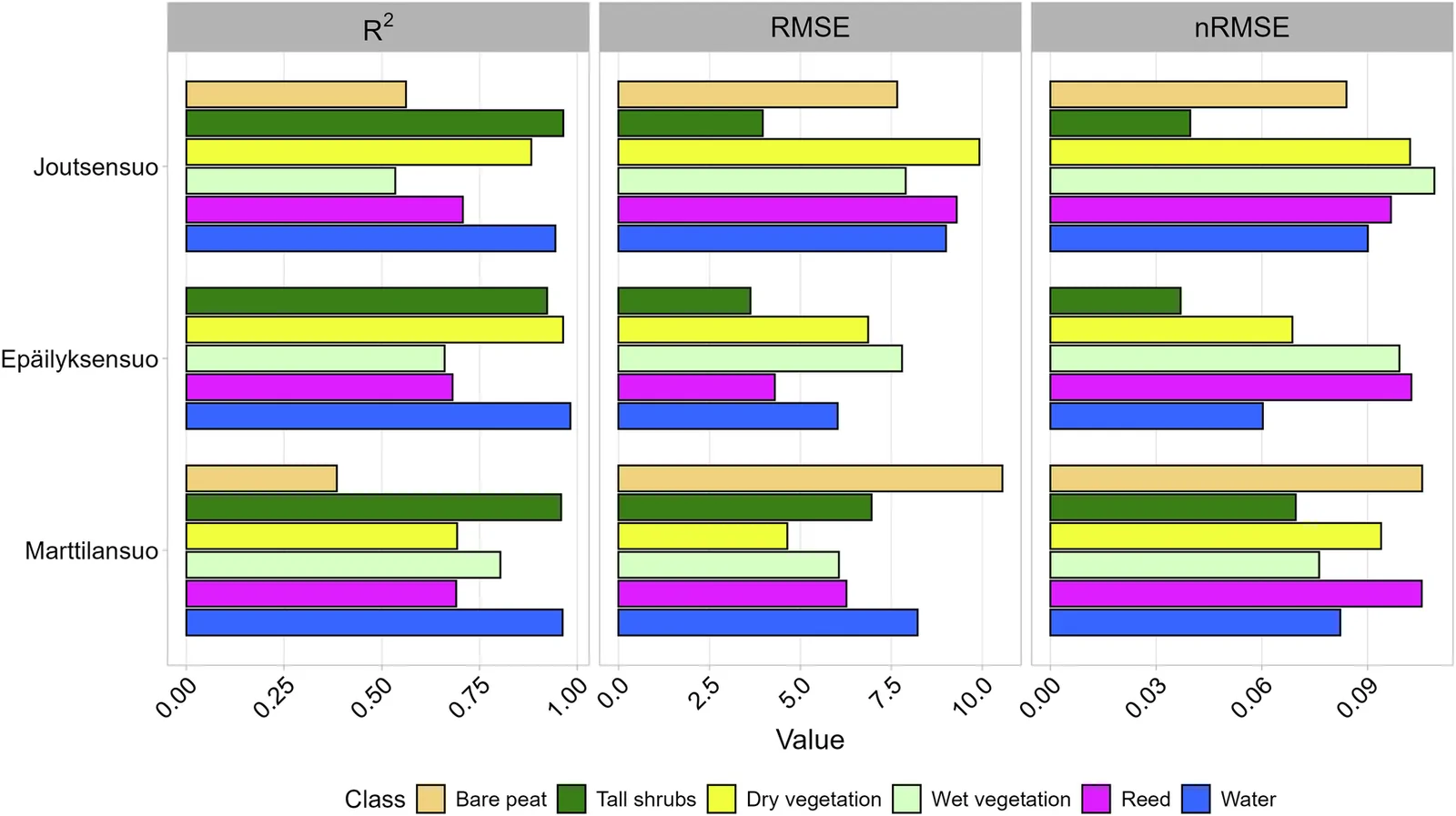
The calculated performance metrics from extreme gradient boosting regression models used for upscaling the surface type %-covers across the study area. Observed-predicted scatter plots can be found from Supplementary material Fig. S5

Multitemporal satellite and CHM-based regressions had mostly a relatively good performance (*R*^2^ = 0.38–0.98, RMSE = 3.63–10.56, nRMSE = 0.04–0.11; Fig. 5). Water (R^2^ = 0.94–0.98, RMSE = 6.03–9.01, nRMSE = 0.06–0.09) and tall shrubs (R^2^ = 0.92–0.96, RMSE = 3.63–6.96, nRMSE = 0.04–0.07) models performed the best across the sites, while bare peat, reed, and wet vegetation models had in general the lowest performances. The dry vegetation model performed well in Jousensuo and Epäilyksensuo but worse in Marttilansuo.

**Fig. 6:**
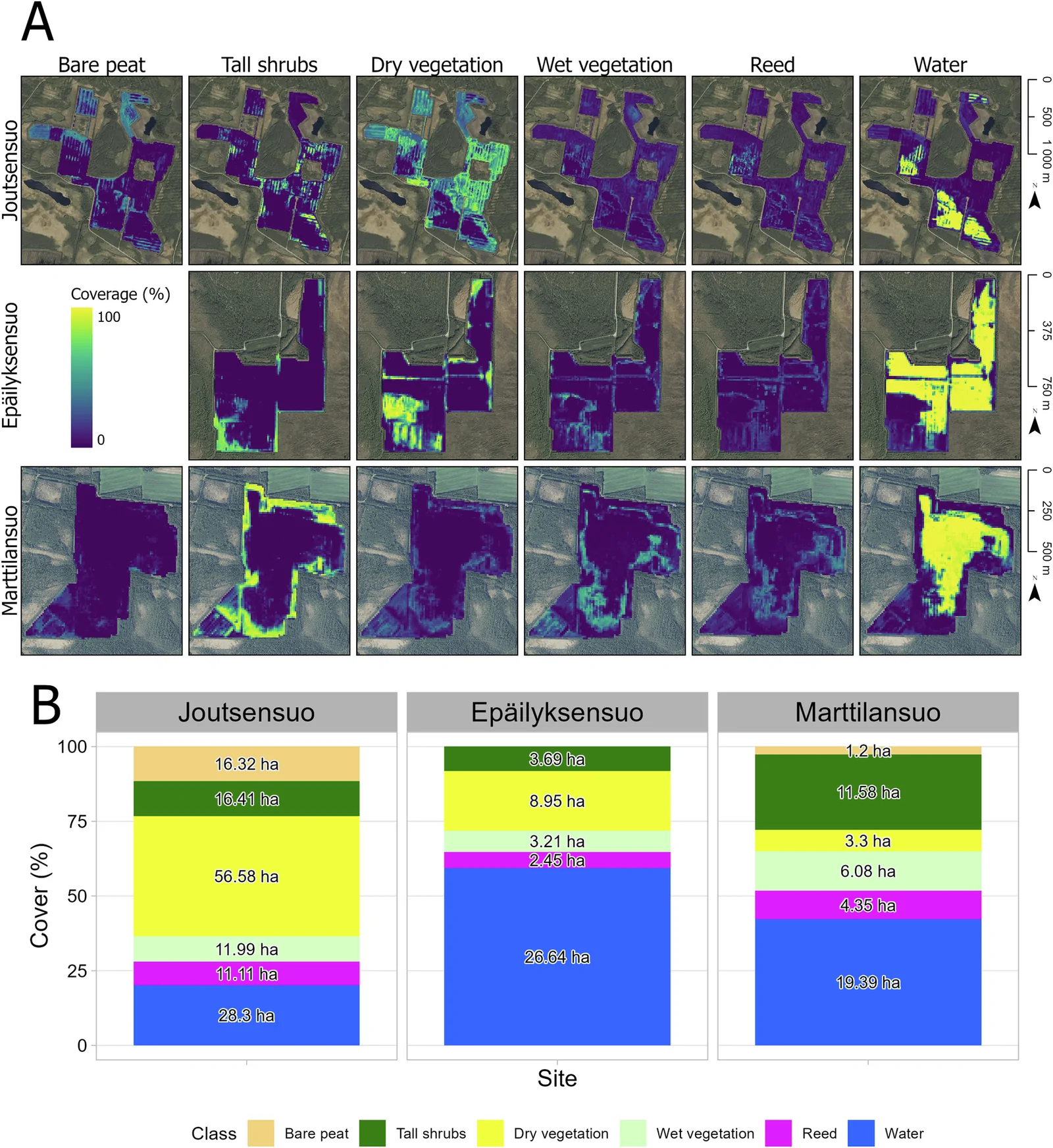
Satellite imagery-based predictions of surface type %-covers for Joutsensuo (first row), Epäilyksensuo (second row) and Marttilansuo (third row) (**A**) and summed areas for each site (**B**). The class bare peat is missing from Epäilyksensuo as it did not exist in the site. Aerial images in the background are open data from National Land Survey of Finland

Based on prediction maps, in all sites, the %-covers followed a gradient of dry vegetation–wet vegetation–reed–water (Fig. 6). Spatial predictions looked credible although predicted reed %-cover in Joutsensuo was not as high as in the training area. Moreover, the bare peat cover was underestimated in the southwestern part of the site (see Figs. 1 and 6). The areal extent of the different surface types differed largely between the sites (Fig. 6B). For example, bare peat had a relatively high total %-cover in the most recently rewetted site Joutsensuo (around 12%), while in the two other sites, it had negligible %-cover.

### Summed Greenhouse Gas Emissions

Measured GHG fluxes showed variation among surface types. Open water consistently exhibited the lowest *R*_eco_ (0.02–2.33 g CO_2_-C m^–2^ d^–1^) and CH₄ fluxes (0.00–0.04 g CH_4_-C m^–2^ d^–1^; Table S4–S5) while highest *R*_eco_ was measured from dry (0.26–8.75 g CO_2_-C m^–2^ d^–1^) and wet vegetation (0.44–7.23 g CO_2_-C m^–2^ d^–1^). CH₄ fluxes were highest on reed (0.01–0.59 g CH_4_-C m^–2^ d^–1^), dry vegetation (0.02–1.11 g CH_4_-C m^–2^ d^–1^), and wet vegetation (0.02–0.47 g CH_4_-C m^–2^ d^–1^). In contrast, N₂O fluxes highly varied temporally and spatially and showed no consistent pattern among surface types (Table S6).

Mann–Whitney U tests indicated that *R*_eco_ differed between most class pairs with statistical significance (*p* value < 0.05). Dry and wet vegetation had the smallest difference with statistical significance found only in Marttilansuo (Fig. S1). CH_4_ fluxes differed similarly but statistical significances were not found between dry vegetation and reed in Epäilyksensuo, and dry and wet vegetation in Marttilansuo (Fig. S2). N_2_O fluxes did not differ statistically significantly from each other across most class pairs (Fig. S3). Kruskal-Wallis tests showed statistically significant difference between the classes for each site and GHG (Figs. S1–S3).

**Fig. 7:**
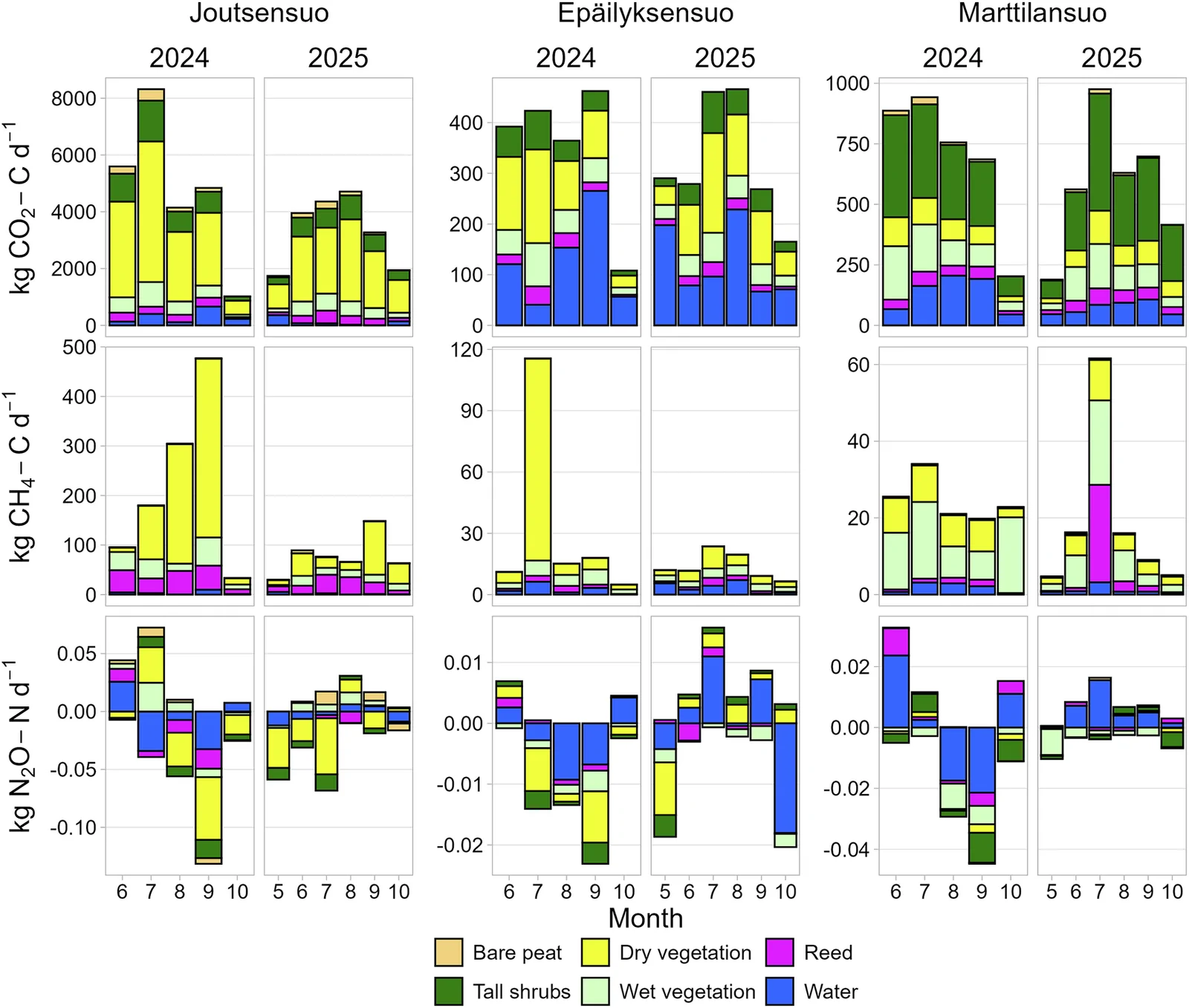
Summed ecosystem respiration (CO_2_-C), CH_4_-C and N_2_O-N fluxes per measurement moment across the entire study sites. Bars represent total fluxes, and stacked colours show the contribution of different surface types. Tall shrubs sums are based on coefficients from dry vegetation for CO_2_ and N_2_O and based on constant coefficient from Buzacott et al. (2025) for CH_4_

Upscaled GHG flux sums showed high spatial and temporal variation across the study sites (Fig. 7). *R*_eco_ was generally the highest in July and lowest in October. Similarly, CH_4_ fluxes followed similar seasonal trend, but in Joutsensuo, the highest total CH_4_ emissions occurred in September across both growing seasons. N_2_O fluxes showed the highest variation within the growing seasons, with sites alternating between a net source and a net sink depending on the measurement moment.

**Fig. 8:**
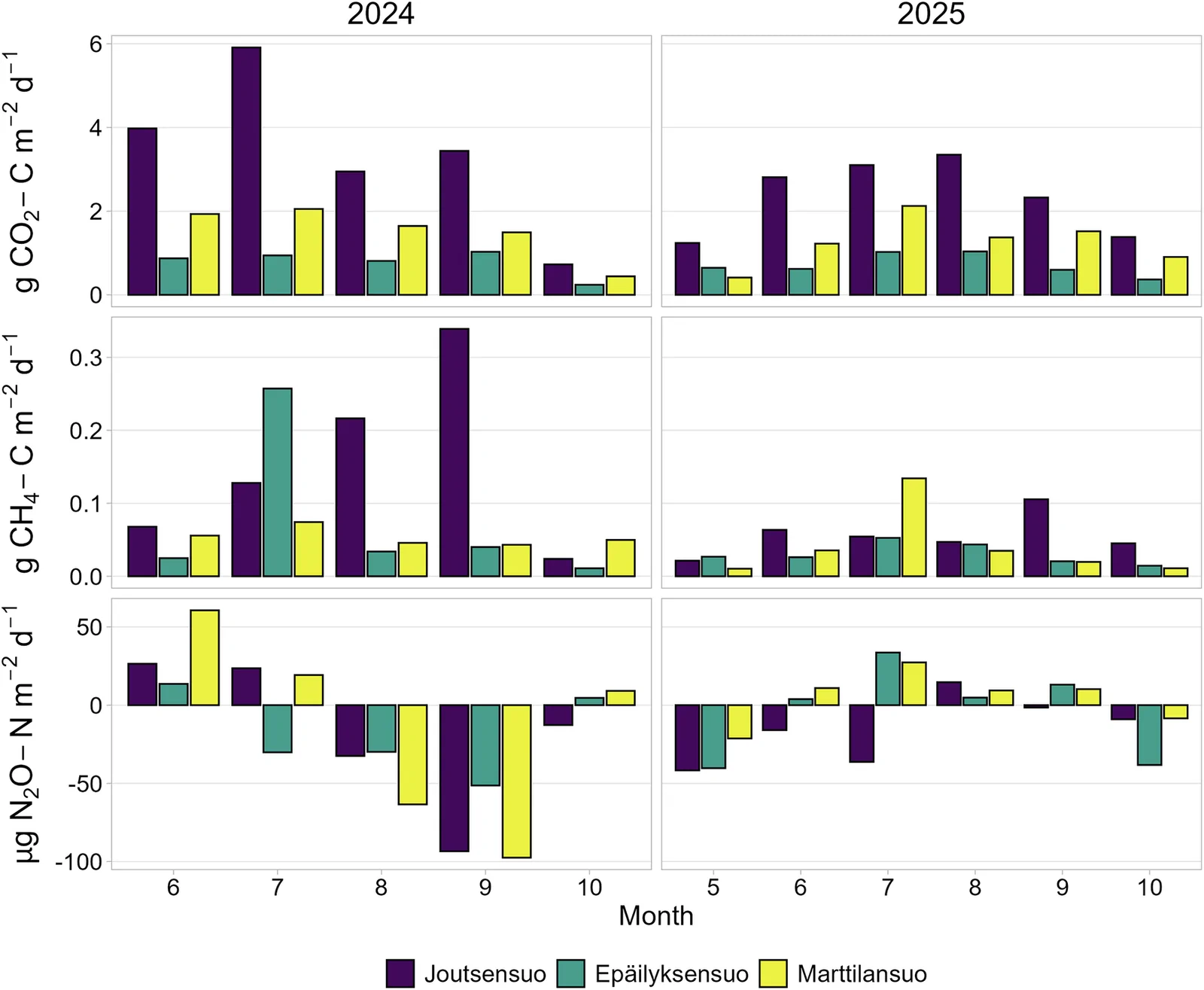
Area normalised means for ecosystem respiration (CO_2_-C), CH_4_-C and N_2_O-N fluxes across the entire study sites at different measurement moments. Emission factors for each surface type per site and month are in supplementary material (Tables S4–S6)

When upscaled GHG fluxes were normalised by site area, Joutsensuo had the highest *R*_eco_ (0.73–5.91 g CO_2_-C m^–2^ d^–1^) and CH_4_ fluxes (0.02–0.34 g CH_4_-C m^–2^ d^–1^) across the two growing seasons (Fig. 8). Epäilyksensuo had the lowest *R*_eco_ (0.24–1.04 g CO_2_-C m^–2^ d^–1^) and Marttilansuo had the lowest CH_4_ (0.01–0.13 g CH_4_-C m^–2^ d^–1^). N_2_O fluxes varied between –98 and 61 μg N_2_O-N m^–2^ d^–1^ with all the sites having similar temporal patterns across the two growing seasons.

## Discussion

### Drones as a Bridge Between Field Data and Satellite Imagery

We have shown that drone-derived high-resolution classification maps and satellite imagery can be used to upscale wetland surface type %-covers outside the drone-covered areas with a relatively good accuracy. Furthermore, the remote sensing-derived surface type areas can be used to quantify momentary GHG fluxes. While previous studies have upscaled GHG fluxes using remote sensing-based classifications, they have mainly utilised a coarse approach by assigning one surface type class to each pixel in medium resolution satellite imagery (e.g. Ludwig et al. 2024) or spatially limited areas with high-resolution imagery (e.g. Sjögersten et al. 2023).

**Fig. 9:**
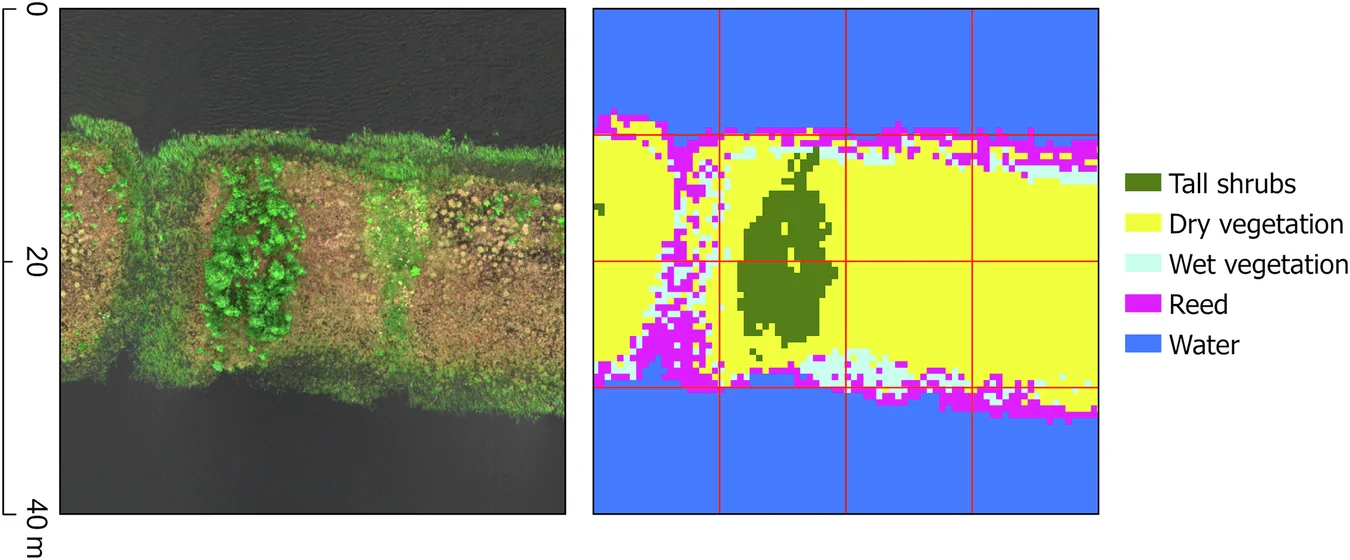
Example of Sentinel-2 pixels (red grid) containing several surface types in Epäilyksensuo. RGB-presentation of Altum-PT orthomosaic in the left and classified surface types in the right

We argue that surface type %-cover approach is superior to one-class-per-one-pixel approach when assessing GHG fluxes over large areas that cannot be fully covered by drone flights. Nevertheless, one-class-per-one-pixel approach can be sufficient in areas where spatial heterogeneity is low and the surface types cover large and continuous areas. In our study, multiple surface types were apparent in a single Sentinel-2 pixel. Additionally, some surface types can be highly underrepresented in the mapped area and can occupy only a small part of Sentinel-2 pixels, which would lead to a non-presence in the site if a pixel-based classification would be conducted. For instance, the reed class occupied only a small proportion in several Sentinel-2 pixels in Epäilyksensuo (see Fig. 9).

Upscaling from drone to satellite level functions properly if the %-cover variation is captured well in the training data. In our case, we had to digitize more training data for Marttilansuo bare peat regression model due to lack of high bare peat %-cover Sentinel-2 pixels in drone mapped area. While this improved the bare peat %-cover model performance, the model still lacked enough data, which led to underestimation of peat cover in the southwestern part of the site. In future studies, the drone-covered area should be planned so that it captures the variation within all focal surface types.

We used a 50 cm spatial resolution grid in our upscaling. The grid corresponds approximately to the areal extent of the used chambers (60 cm × 60 cm) and is also in line with an optimal spatial resolution for characterising surface patterns in highly heterogeneous wetland ecosystems (Räsänen and Virtanen 2019; Steenvoorden and Limpens 2023). Earlier studies have also discussed that object-based image analysis methods should be used in cases where pixel size is much smaller than the size of the objects of interest (Blaschke et al. 2014), i.e. surface type patches in our case. While our 0.25 m^2^ sized pixels are still smaller than typical surface type patches, they are notably larger than the original ~0.025 m^2^ pixels in the drone data and thus avoid the problem of random noise typical to pixel-based classifications (Blaschke et al. 2014) and object-based classifications with very small segments (Räsänen and Virtanen 2019). Furthermore, earlier studies have indicated that if segment size in object-based methods is too large, classification accuracy is decreased (Räsänen and Virtanen 2019; Steenvoorden and Limpens 2023); therefore, the segmentation of the 50 cm pixels into objects could have led to less accurate maps.

We could have used other, higher spatial resolution, satellite datasets, such as PlanetScope (spatial resolution of 3 m; Planet Team (2024)) for our upscaling purposes. While Bergamo et al. (2023) used PlanetScope to upscale *Rosa Rugosa* %-cover in Estonian coastal habitats, Riihimäki et al. (2019) and Keränen et al. (2024) showed that Sentinel-2 can offer better fractional vegetation and flark %-cover estimations compared to PlanetScope, probably due to latter’s lower radiometric quality (Frazier and Hemingway 2021). Nevertheless, image fusion of PlanetScope and Sentinel-2 have provided increased performance in landcover classification when compared to both datasets alone (Zhao et al. 2026), and the methodology could have been utilised in this study as well.

We used random forest and XGBoost for our classification and regression tasks, respectively, but we could have tested more sophisticated methods. Random forest often performs relatively well when compared to other machine learning algorithms in remote sensing tasks (Belgiu and Drăguţ 2016), but some studies have shown that deep neural networks can have a slight edge on performance (Sharma et al. 2024; Ball et al. 2026). Furthermore, convolutional neural networks have been broadly tested in remote sensing tasks and have shown superior performance in cases when spatial context matter (Kattenborn et al. 2021). In our case convolutional neural network approach might have decreased misclassification of reed in areas where open water does not exist, such as drier areas in Joutsensuo. Furthermore, recent development in remote sensing foundation models, i.e. pre-trained models that have been constructed from multisource geospatial big data have shown promise (Lu et al. 2025). Recent studies have shown mixed evidence of foundation models, with one study showing improved performance compared to multitemporal satellite imagery (Ball et al. 2026) while another case showing that the better performance in not guaranteed (Rojas Lucero et al. 2026). Therefore, future studies could test whether foundation models could be used for upscaling drone-based classifications more efficiently than multitemporal Sentinel-2 imagery and airborne LiDAR data.

### Spatiotemporal Variation Within Greenhouse Gas Emissions

We observed high spatial and temporal variation in GHG fluxes between the surface types and sites. Additionally, we were able to derive emission factors that capture site-specific total emissions more realistically than the chamber observations alone. Our area normalized *R*_eco_ (0.24–5.91 g CO_2_-C m^–2^ d^–1^) were comparable with earlier studies from rewetted peat extraction sites (0.49–8.67 g CO_2_-C m^–2^ d^–1^; (Tuittila et al. 1999; Minke et al. 2016)). In our study, open water had lowest *R*_eco_, and the total open water %-cover strongly influenced the overall site-level emissions. In contrast, the highest *R*_eco_ were measured from dry and wet vegetation depending on the site, likely reflecting the variation in dominating vegetation and soil conditions between sites. Joutsensuo had the highest *R*_eco_ in both study years, which was due to smaller open water area and larger proportion of dry vegetation compared to the other sites.

All sites were CH_4_ sources throughout the growing seasons with emissions varying between sites and measurement moments. The area normalised CH_4_ fluxes (0.01–0.41 g CH_4_-C m^–2^ d^–1^) were generally within the range of previous observations from rewetted peat extraction sites (0.02–0.37 g CH_4_-C m^–2^ d^–1^; Minke et al. 2016; Jordan et al. 2020; Aitova et al. 2023; Mander et al. 2023). However, fluxes in our study were notably higher than fluxes reported by e.g. Tuittila et al. (2000) for a recently rewetted Finnish cutover fen (<0.01 g CH_4_-C m^–2^ d^–1^) likely because methanogenic communities at their site had not yet fully developed after rewetting. Similar to *R*_eco_, CH_4_ fluxes were lowest in open water whereas the highest emissions, depending on the site, were measured from reed, dry vegetation, and wet vegetation likely due to plant-mediated CH₄ transport and better substrate availability (Minke et al. 2016; Ge et al. 2024). Among the sites, Joutsensuo had the highest area-normalised fluxes due to its lower open water %-cover and higher emissions from dry vegetation which occupied large areas within the site. However, our measurements only accounted for diffusive CH_4_ fluxes while ebullition from water surfaces were not included which may cause some underestimation of the total emissions.

N_2_O fluxes were close to zero, with sites alternating between weak sources and sinks, similar to previously reported fluxes from rewetted peat extraction sites (Minke et al. 2016; Jordan et al. 2020; Aitova et al. 2023; Mander et al. 2023). No consistent patterns were observed between the surface types (Table S6). N_2_O emissions are known to exhibit high spatial and temporal variability, often characterized by short-lived emission peaks that are difficult to predict (Minke et al. 2016; Jordan et al. 2020) and that require continuous monitoring. This introduces considerable uncertainty when upscaling fluxes is based on a limited number of manual chamber measurements that are conducted around once a month. Therefore, in a way, our upscaled results are snapshots from certain moments across two growing seasons. Furthermore, the measured N_2_O fluxes were mostly smaller than reported accuracy of the equipment (around ±121 μg N_2_O-N m^–2^ d^–1^; Table S6), hence it is possible that measured fluxes were essentially noise. Nevertheless, the overall contribution of N_2_O emissions to the whole GHG exchange was minor.

Our GHG emission estimates have some uncertainties due to low number of plots per site. Even though we chose the measurement locations carefully, it is impossible to assess whether our plots represent the average conditions for each surface type well. Therefore, the calculated GHG sums and fluxes are indicative at best. The highest uncertainties in our data are probably in the dry vegetation measurements in Joutsensuo, as its measured CH_4_ fluxes were substantially higher than in other surface types and sites. One plausible reason for this behaviour might be that the location of our dry vegetation measurements can have been used as a landfill for soil and other material during the rewetting of the site. Therefore, the soil properties can be largely different compared to the other areas of the site, which affects the CH_4_ fluxes (Ge et al. 2024). Additionally, we did not have measurements within tall shrub areas which increases the uncertainty of GHG sums across the sites as we used the CH_4_ coefficients from Buzacott et al. (2025) and dry vegetation coefficients for CO_2_ and N_2_O as reference. In Joutsensuo and Epäilyksensuo, the %-cover of tall shrubs is relatively small but in Marttilansuo it has larger %-cover (Fig. 6), which means that if dry vegetation CH_4_ coefficients were used as reference, the total CH_4_-C sum would be almost double than the ones presented here (Fig. 7). However, as forested peat extraction areas usually emit minor CH_4_ emissions (Mäkiranta et al. 2007; Bravo et al. 2018; Buzacott et al. 2025), we think that literature-based coefficient represent the CH_4_ emissions better than our own field measurements from open vegetation areas.

More ecologically realistic surface types could have been produced using a floristic analysis based on vegetation inventories and clustering (e.g. Räsänen et al. 2020; Wolff et al. 2025) after which GHG plots could have been placed in a way that they better represent the ecological gradient within the sites. However, our statistical tests indicated that our classes mostly differed from each other based on GHG data (Figs. S2–S4), and the vegetation structure also differed (Fig. S1). In that sense, we were able to capture spatiotemporal variation among GHGs within the sites.

When comparing the time from rewetting, there is no clear pattern between the sites. In the case of surface types reed and water, it seems that the measured *R*_eco_ and CH_4_ fluxes followed the time pattern, i.e. highest fluxes are measured from the most recently rewetted site and the lowest fluxes from the oldest rewetted site (Table S5) which could be resulted from turnover of labile carbon sources (e.g. Fenner et al. 2011; Evans et al. 2016; Darusman et al. 2023). However, as the site and chamber points are limited, this cannot be concluded from this study alone. Additionally, these two surface types were the only ones that were very similar to each other across all sites, while the plant functional type cover structure varied more among the other types between the sites (Fig. S1). Long-term monitoring or a chronosequence with a large number of sites would be needed to properly assess how the GHG fluxes develop over time in rewetted peat extraction sites. As a final remark, we measured only *R*_eco_; therefore, no conclusions can be drawn about changes in the net ecosystem carbon balance of the sites with time since rewetting.

## Conclusion

We tested a drone-to-satellite surface type cover upscaling approach to quantify chamber-measured *R*_eco_, CH_4_ exchange, and N_2_O exchange from rewetted peat extraction sites. Machine learning-based classifiers and models produced credible area estimates for different surface types, which were used for upscaling the field-measured GHG fluxes. Particularly, the drone-based classification was successfully used as a mediator between field measurements covering small areas and notably larger Sentinel-2 satellite image pixels. The GHG fluxes showed high spatiotemporal variation across two growing seasons with emission peaks in July. Additionally, high water and reed %-cover reduced overall emissions due to higher emissions in drier surface types but due to lack of gross primary production estimations, no conclusion can be drawn about net ecosystem carbon balances. The proposed approach can be used in other upscaling purposes where there is a need to link point-based measurements to satellite image pixels.

## Supplementary information

Supplementary information

## Data Availability

Data will be made available on request.

## References

[CR1] Aitova E, Morley T, Wilson D, Renou-Wilson F (2023) A review of greenhouse gas emissions and removals from Irish peatlands. Mires Peat 29:04 10.19189/MAP.2022.SNPG.STA.2414.

[CR2] Ball JG et al. (2026) Geospatial foundation models enable data-efficient tree species mapping in temperate mountain forests 10.1016/j.srs.2026.100466.

[CR3] Barnes EM, Clarke TR, Richards SE, et al. (2000) Coincident detection of crop water stress, nitrogen status and canopy density using ground-based multispectral data. Bloomington, Minnesota, USA

[CR4] Belgiu M, Drăguţ L (2016) Random forest in remote sensing: A review of applications and future directions. ISPRS J Photogramm Remote Sens 114:24–31 10.1016/j.isprsjprs.2016.01.011.

[CR5] Bergamo TF, De Lima RS, Kull T et al. (2023) From UAV to PlanetScope: Upscaling fractional cover of an invasive species Rosa rugosa. J Environ Manag 336:117693 10.1016/j.jenvman.2023.117693. 36913856

[CR6] Blaschke T, Hay GJ, Kelly M et al. (2014) Geographic object-based image analysis – Towards a new paradigm. ISPRS J Photogramm Remote Sens 87:180–191 10.1016/j.isprsjprs.2013.09.014. PMC3945831 24623958

[CR7] Bravo TG, Rochefort L, Strack M (2018) The impact of a black spruce (*Picea mariana*) plantation on carbon exchange in a cutover peatland in Western Canada. Can J Res 48:388–398 10.1139/cjfr-2017-0378.

[CR8] Breiman L (2001) Random Forests. Mach Learn 45:5–32 10.1023/A:1010933404324.

[CR9] Brenning A (2012) Spatial cross-validation and bootstrap for the assessment of prediction rules in remote sensing: The R package sperrorest. In: 2012 IEEE International Geoscience and Remote Sensing Symposium. IEEE, Munich, Germany, pp 5372–5375

[CR10] Buzacott AJV, Laasasenaho K, Lauhanen R et al. (2025) Afforestation-related fertilisation quickly turns barren cutaway peatland into a carbon dioxide sink. Glob Change Biol 31:e70644 10.1111/gcb.70644. PMC12710595 41405440

[CR11] Chapman S, Buttler A, Francez A-J et al. (2003) Exploitation of northern peatlands and biodiversity maintenance: a conflict between economy and ecology. Front Ecol Environ 1:525–532 10.1890/1540-9295.

[CR12] Chen T, Guestrin C (2016) XGBoost: A scalable tree boosting system. In: *Proceedings of the 22nd ACM SIGKDD International Conference on Knowledge Discovery and Data Mining*. ACM, San Francisco California USA, pp 785–794

[CR13] Darusman T, Murdiyarso D, Impron, Anas I (2023) Effect of rewetting degraded peatlands on carbon fluxes: a meta-analysis. Mitig Adapt Strateg Glob Change 28:10 10.1007/s11027-023-10046-9.

[CR14] Davidson S, Santos M, Sloan V et al. (2017) Upscaling CH4 fluxes using high-resolution imagery in arctic tundra ecosystems. Remote Sens 9:1227 10.3390/rs9121227.

[CR15] Evans CD, Renou-Wilson F, Strack M (2016) The role of waterborne carbon in the greenhouse gas balance of drained and re-wetted peatlands. Aquat Sci 78:573–590 10.1007/s00027-015-0447-y.

[CR16] Fenner N, Williams R, Toberman H et al. (2011) Decomposition ‘hotspots’ in a rewetted peatland: implications for water quality and carbon cycling. Hydrobiologia 674:51–66 10.1007/s10750-011-0733-1.

[CR17] Frazier AE, Hemingway BL (2021) A technical review of planet smallsat data: Practical considerations for processing and using PlanetScope imagery. Remote Sens 13:3930 10.3390/rs13193930.

[CR18] Gao B (1996) NDWI—A normalized difference water index for remote sensing of vegetation liquid water from space. Remote Sens Environ 58:257–266 10.1016/S0034-4257(96)00067-3.

[CR19] Ge M, Korrensalo A, Laiho R et al. (2024) Plant-mediated CH4 exchange in wetlands: A review of mechanisms and measurement methods with implications for modelling. Sci Total Environ 914: 169662 10.1016/j.scitotenv.2023.169662. 38159777

[CR20] Hashemi J, Räsänen A, Virtanen T et al. (2025) Coarse land cover datasets bias Arctic-Boreal wetland methane budgets. Commun Earth Environ 6:903 10.1038/s43247-025-02963-1. PMC12618245 41245527

[CR21] Huete AR (1988) A soil-adjusted vegetation index (SAVI). Remote Sens Environ 25:295–309 10.1016/0034-4257(88)90106-X.

[CR22] Isoaho A, Elo M, Marttila H et al. (2024a) Monitoring changes in boreal peatland vegetation after restoration with optical satellite imagery. Sci Total Environ 957:177697 10.1016/j.scitotenv.2024.177697. 39579908

[CR23] Isoaho A, Ikkala L, Päkkilä L et al. (2024b) Multi-sensor satellite imagery reveals spatiotemporal changes in peatland water table after restoration. Remote Sens Environ 306:114144 10.1016/j.rse.2024.114144.

[CR24] Itoh M, Okimoto Y, Hirano T, Kusin K (2017) Factors affecting oxidative peat decomposition due to land use in tropical peat swamp forests in Indonesia. Sci Total Environ 609:906–915 10.1016/j.scitotenv.2017.07.132. 28783903

[CR25] Ivanova K, Virkkala A-M, Brovkin V et al. (2026) High-resolution remote sensing and machine-learning-based upscaling of methane fluxes: a case study in the Western Canadian tundra. Biogeosciences 23:233–262 10.5194/bg-23-233-2026.

[CR26] Joosten H, Clarke D (2002) Wise use of Mires and Peatlands - Background and Principles including a Framework for Decision-making. International Mire Conservation Group and International Peat Society

[CR27] Joosten H, Sirin A, Couwenberg J, et al. (2016) The role of peatlands in climate regulation. In: Bonn A, Allott T, Evans M, et al. (eds) Peatland Restoration and Ecosystem Services. Cambridge University Press, Cambridge, pp 63–76

[CR28] Jordan S, Strömgren M, Fiedler J et al. (2020) Methane and Nitrous Oxide Emission Fluxes Along Water Level Gradients in Littoral Zones of Constructed Surface Water Bodies in a Rewetted Extracted Peatland in Sweden. Soil Syst 4:17 10.3390/soilsystems4010017.

[CR29] Kattenborn T, Leitloff J, Schiefer F, Hinz S (2021) Review on Convolutional Neural Networks (CNN) in vegetation remote sensing. ISPRS J Photogramm Remote Sens 173:24–49 10.1016/j.isprsjprs.2020.12.010.

[CR30] Kelly J, Kljun N, Eklundh L et al. (2021) Modelling and upscaling ecosystem respiration using thermal cameras and UAVs: Application to a peatland during and after a hot drought. Agric Meteorol 300: 108330 10.1016/j.agrformet.2021.108330.

[CR31] Keränen K, Isoaho A, Räsänen A et al. (2024) Multi-resolution remote sensing for flark area detection in boreal aapa mires. Int J Remote Sens 45:4324–4343 10.1080/01431161.2024.2359732.

[CR32] Khan AA, Chaudhari O, Chandra R (2024) A review of ensemble learning and data augmentation models for class imbalanced problems: Combination, implementation and evaluation. Expert Syst Appl 244:122778 10.1016/j.eswa.2023.122778.

[CR33] Klehr D, Stoffels J, Hill A et al. (2025) Mapping tree species fractions in temperate mixed forests using Sentinel-2 time series and synthetically mixed training data. Remote Sens Environ 323:114740 10.1016/j.rse.2025.114740.

[CR34] Kozlov SA, Lundin L, Avetov NA (2016) Revegetation dynamics after 15 years of rewetting in two extracted peatlands in Sweden. Mires Peat 18:5 10.19189/MaP.2015.OMB.204.

[CR35] Lang M, Binder M, Richter J et al. (2019) mlr3: A modern object-oriented machine learning framework in R. J Open Source Softw 4:1903 10.21105/joss.01903.

[CR36] Lehmann J, Münchberger W, Knoth C et al. (2016) High-resolution classification of South Patagonian Peat Bog microforms reveals potential gaps in up-scaled CH4 fluxes by use of unmanned aerial system (UAS) and CIR imagery. Remote Sens 8:173 10.3390/rs8030173.

[CR37] Leifeld J, Menichetti L (2018) The underappreciated potential of peatlands in global climate change mitigation strategies. Nat Commun 9: 1071 10.1038/s41467-018-03406-6. PMC5851997 29540695

[CR38] Liu HQ, Huete A (1995) A feedback based modification of the NDVI to minimize canopy background and atmospheric noise. IEEE Trans Geosci Remote Sens 33:457–465 10.1109/TGRS.1995.8746027.

[CR39] Lu S, Guo J, Zimmer-Dauphinee JR et al. (2025) Vision Foundation Models in Remote Sensing: A survey. IEEE Geosci Remote Sens Mag 13:190–215 10.1109/MGRS.2025.3541952.

[CR40] Ludwig SM, Schiferl L, Hung J et al. (2024) Resolving heterogeneous fluxes from tundra halves the growing season carbon budget. Biogeosciences 21:1301–1321 10.5194/bg-21-1301-2024.

[CR41] Lundin L, Nilsson T, Jordan S et al. (2017) Impacts of rewetting on peat, hydrology and water chemical composition over 15 years in two finished peat extraction areas in Sweden. Wetl Ecol Manag 25:405–419 10.1007/s11273-016-9524-9.

[CR42] Mahoney MJ, Johnson LK, Silge J, et al. (2023) Assessing the performance of spatial cross-validation approaches for models of spatially structured data. arXiv preprint arXiv:2303.07334. 10.48550/arXiv.2303.07334.

[CR43] Mäkiranta P, Hytönen J, Aro L et al. (2007) Soil greenhouse gas emissions from afforested organic soil croplands and cutaway peatlands. Boreal Environ Res 12:159–175.

[CR44] Mander Ü, Espenberg M, Melling L, Kull A (2023) Peatland restoration pathways to mitigate greenhouse gas emissions and retain peat carbon. Biogeochemistry 167:523–543 10.1007/s10533-023-01103-1. PMC11068583 38707516

[CR45] McFeeters SK (1996) The use of the Normalized Difference Water Index (NDWI) in the delineation of open water features. Int J Remote Sens 17:1425–1432 10.1080/01431169608948714.

[CR46] Meinshausen N (2006) Quantile Regression Forests. J Mach Learn Res 7:983–999.

[CR47] Minke M, Augustin J, Burlo A et al. (2016) Water level, vegetation composition, and plant productivity explaingreenhouse gas fluxes in temperate cutover fens after inundation. Biogeosciences 13:3945–3970 10.5194/bg-13-3945-2016.

[CR48] Planet Team (2024) Planet Application Program Interface: In Space for Life on Earth.

[CR49] Probst P, Wright MN, Boulesteix A (2019) Hyperparameters and tuning strategies for random forest. WIREs Data Min Knowl Discov 9:e1301 10.1002/widm.1301.

[CR50] Putkiranta P, Juutinen S, Räsänen A et al. (2025) Seasonal quantification of aquatic macrophytes in small boreal lakes with multiscale remote sensing. Sci Total Environ 1000:180333 10.1016/j.scitotenv.2025.180333. 40914117

[CR51] Ramchunder SJ, Brown LE, Holden J (2012) Catchment-scale peatland restoration benefits stream ecosystem biodiversity. J Appl Ecol 49:182–191 10.1111/j.1365-2664.2011.02075.x.

[CR52] Räsänen A, Albrecht E, Annala M et al. (2023) After-use of peat extraction sites – A systematic review of biodiversity, climate, hydrological and social impacts. Sci Total Environ 882:163583 10.1016/j.scitotenv.2023.163583. 37086986

[CR53] Räsänen A, Aurela M, Juutinen S et al. (2020) Detecting northern peatland vegetation patterns at ultra-high spatial resolution. Remote Sens Ecol Conserv 6:457–471 10.1002/rse2.140.

[CR54] Räsänen A, Manninen T, Korkiakoski M et al. (2021) Predicting catchment-scale methane fluxes with multi-source remote sensing. Landsc Ecol 36:1177–1195 10.1007/s10980-021-01194-x.

[CR55] Räsänen A, Virtanen T (2019) Data and resolution requirements in mapping vegetation in spatially heterogeneous landscapes. Remote Sens Environ 230:111207 10.1016/j.rse.2019.05.026.

[CR56] Reddin E, Hanafin J, Tong M et al. (2025) Modelling water table depth at rewetted peatlands with Sentinel-1 and Sentinel-2. Sci Remote Sens 11:100238 10.1016/j.srs.2025.100238.

[CR57] Riihimäki H, Luoto M, Heiskanen J (2019) Estimating fractional cover of tundra vegetation at multiple scales using unmanned aerial systems and optical satellite data. Remote Sens Environ 224:119–132 10.1016/j.rse.2019.01.030.

[CR58] Rodriguez-Galiano VF, Ghimire B, Rogan J et al. (2012) An assessment of the effectiveness of a random forest classifier for land-cover classification. ISPRS J Photogramm Remote Sens 67:93–104 10.1016/j.isprsjprs.2011.11.002.

[CR59] Rojas Lucero JC, Kolarik N, Brandt J, et al. (2026) Spectral indices outperform AlphaEarth foundation embeddings for aboveground biomass estimation in tropical Andean Forests. EarthArXiv. 10.31223/X58X7V.

[CR60] Sadeghi M, Jones SB, Philpot WD (2015) A linear physically-based model for remote sensing of soil moisture using short wave infrared bands. Remote Sens Environ 164:66–76 10.1016/j.rse.2015.04.007.

[CR61] Sallinen A, Tuominen S, Kumpula T, Tahvanainen T (2019) Undrained peatland areas disturbed by surrounding drainage: a large scale GIS analysis in Finland with a special focus on aapa mires. Mires Peat 24:38 10.19189/MAP.2018.AJB.391.

[CR62] Sharma V, Honkavaara E, Hayden M, Kant S (2024) UAV remote sensing phenotyping of wheat collection for response to water stress and yield prediction using machine learning. Plant Stress 12:100464 10.1016/j.stress.2024.100464.

[CR63] Sjögersten S, Ledger M, Siewert M et al. (2023) Optical and radar Earth observation data for upscaling methane emissions linked to permafrost degradation in sub-Arctic peatlands in northern Sweden. Biogeosciences 20:4221–4239 10.5194/bg-20-4221-2023.

[CR64] Steenvoorden J, Bartholomeus H, Limpens J (2023) Less is more: Optimizing vegetation mapping in peatlands using unmanned aerial vehicles (UAVs). Int J Appl Earth Obs Geoinf 117:103220 10.1016/j.jag.2023.103220.

[CR65] Steenvoorden J, Limpens J (2023) Upscaling peatland mapping with drone-derived imagery: impact of spatial resolution and vegetation characteristics. GIScience Remote Sens 60:2267851 10.1080/15481603.2023.2267851.

[CR66] Syke (2023) Peat production areas with after-use category dataset

[CR67] Tou JT, Gonzales RC (1974) Pattern recognition principles. Addison-Wesley Publishing Company

[CR68] Tucker CJ (1979) Red and photographic infrared linear combinations for monitoring vegetation. Remote Sens Environ 8:127–150 10.1016/0034-4257(79)90013-0.

[CR69] Tuittila E, Komulainen V, Vasander H et al. (2000) Methane dynamics of a restored cut-away peatland. Glob Change Biol 6:569–581 10.1046/j.1365-2486.2000.00341.x.

[CR70] Tuittila E-S, Komulainen V-M, Vasander H, Laine J (1999) Restored cut-away peatland as a sink for atmospheric CO 2. Oecologia 120:563–574 10.1007/s004420050891. 28308307

[CR71] Velthoen J, Dombry C, Cai J-J, Engelke S (2023) Gradient boosting for extreme quantile regression. Extremes 26:639–667 10.1007/s10687-023-00473-x.

[CR72] Villoslada M, Berner LT, Juutinen S, et al. (2024) Upscaling vascular aboveground biomass and topsoil moisture of subarctic fens from Unoccupied Aerial Vehicles (UAVs) to satellite level. Sci Total Environ 173049 10.1016/j.scitotenv.2024.173049 38735321

[CR73] Vogelmann JE, Rock BN (1986) Assessing forest decline in coniferous forests of Vermont using NS-001 Thematic Mapper Simulator data. Int J Remote Sens 7:1303–1321 10.1080/01431168608948932.

[CR74] Whittington PN, Price JS (2006) The effects of water table draw-down (as a surrogate for climate change) on the hydrology of a fen peatland, Canada. Hydrol Process 20:3589–3600 10.1002/hyp.6376.

[CR75] Wilkinson SL, Andersen R, Moore PA, et al. (2023) Wildfire and degradation accelerate northern peatland carbon release. Nat Clim Change 10.1038/s41558-023-01657-w

[CR76] Wolff F, Kolari THM, Räsänen A et al. (2025) Interannual spectral consistency and spatial uncertainties in UAV -based detection of boreal and subarctic mire plant communities. Remote Sens Ecol Conserv 11:719–739 10.1002/rse2.70017.

[CR77] Wright MN, Ziegler A (2017) ranger: A fast implementation of random forests for high dimensional data in *C++* and *R*. J Stat Softw 77 10.18637/jss.v077.i01

[CR78] Zhao Y, Liu D, Zhu X et al. (2026) RASSFM 2.0: An enhanced M2Msharpening model for PlanetScope–Sentinel-2 image fusion across broad landscapes with improved land cover classification. Remote Sens Environ 338:115371 10.1016/j.rse.2026.115371.

