# Supplementary material for: Upscaling Chamber-measured Greenhouse Gas Emissions in Rewetted Peat Extraction Sites Using Drone and Satellite Imagery
Source: Environ Manage. 2026 Aug 18;76(9):281. doi: 10.1007/s00267-026-02582-2 (PMC13486084; doi:10.1007/s00267-026-02582-2)
Supplement: Supplementary file 1 [file 267_2026_2582_MOESM1_ESM.docx]

**Supplementary material for “Upscaling chamber-measured greenhouse gas emissions in rewetted peat extraction sites using drone and satellite imagery”**

Table S1. Greenhouse gas measurement dates.

| Year | Month | Joutsensuo | Epäilyksensuo | Marttilansuo |
| --- | --- | --- | --- | --- |
| 2024 | 6 | 25.6.2024 | 25.6.2024 | 26.6.2024 |
|  | 7 | 24.7.2024 | 24.7.2024 | 23.7.2024 |
|  | 8 | 20.8.2024 | 20.8.2024 | 21.8.2024 |
|  | 9 | 10.9.2024 | 10.9.2024 | 11.9.2024 |
|  | 10 | 14.10.2024 | 14.10.2024 | 15.10.2024 |
| 2025 | 5 | 20.5.2025 | 20.5.2025 | 21.5.2025 |
|  | 6 | 16.6.2025 | 16.6.2025 | 16.6.2025 |
|  | 7 | 16.7.2025 | 16.7.2025 | 16.7.2025 |
|  | 8 | 12.8.2025 | 12.8.2025 | 13.8.2025 |
|  | 9 | 9.9.2025 | 9.9.2025 | 10.9.2025 |
|  | 10 | 9.10.2025 | 9.10.2025 | 7.10.2025 |

Table S2. Drone and satellite imagery capture dates.

| Dataset | Joutsensuo | Epäilyksensuo | Marttilansuo |
| --- | --- | --- | --- |
| Drone | 18.8.2025 | 8.7.2025 | 9.7.2025 |
| S2 | 11.5.2025 | 11.5.2025 | 11.5.2025 |
| S2 | 13.6.2025 | 13.6.2025 | 13.6.2025 |
| S2 | 13.7.2025 | 15.7.2025 | 13.7.2025 |
| S2 | 11.9.2025 | 6.8.2025 | 5.8.2025 |

Table S3. Tuned hyperparameters for extreme gradient boosting regressions.

| Site | Type | colsample_  bytree | eta | gamma | max_  depth | min_child_  weight | nrounds | subsample |
| --- | --- | --- | --- | --- | --- | --- | --- | --- |
| Joutsensuo | Dry | 0.94 | 0.07 | 1.91 | 9 | 4.66 | 650 | 0.65 |
|  | Bare peat | 0.74 | 0.02 | 0.73 | 4 | 1.73 | 800 | 0.86 |
|  | Reed | 0.93 | 0.07 | 1.72 | 5 | 5.02 | 250 | 0.62 |
|  | Tall shrubs | 0.82 | 0.06 | 1.28 | 3 | 9.91 | 1000 | 0.79 |
|  | Water | 0.73 | 0.02 | 1.51 | 5 | 5.22 | 400 | 0.6 |
|  | Wet | 0.84 | 0.08 | 1.94 | 10 | 3.05 | 350 | 0.66 |
| Epäilyksensuo | Dry | 0.86 | 0.06 | 0.01 | 8 | 2.32 | 950 | 0.5 |
|  | Reed | 0.82 | 0.08 | 1.46 | 3 | 4.44 | 550 | 0.61 |
|  | Tall shrubs | 0.92 | 0.08 | 1.26 | 2 | 1.81 | 650 | 0.76 |
|  | Water | 0.54 | 0.01 | 1.38 | 2 | 5.76 | 950 | 0.69 |
|  | Wet | 0.54 | 0.12 | 0.65 | 6 | 9.87 | 150 | 0.9 |
| Marttilansuo | Dry | 0.89 | 0.05 | 1.23 | 8 | 3.15 | 550 | 0.52 |
|  | Peat | 0.53 | 0.19 | 0.04 | 5 | 3.84 | 50 | 0.76 |
|  | Reed | 0.79 | 0.04 | 2 | 10 | 9.18 | 900 | 0.72 |
|  | Tall shrubs | 0.79 | 0.04 | 0.29 | 7 | 7.40 | 900 | 0.56 |
|  | Water | 0.52 | 0.03 | 1.67 | 9 | 8.89 | 350 | 0.57 |
|  | Wet | 0.82 | 0.07 | 0.19 | 2 | 1.65 | 350 | 0.52 |

Table S4. Emission factors for g CO_2_-C m^-2^ d^-1^ for each surface type. Factors have been rounded to three decimals.

| Site | Year | Time | Bare peat | Dry vegetation | Wet vegetation | Reed | Water |
| --- | --- | --- | --- | --- | --- | --- | --- |
| Joutsensuo | 2024 | 6 | 1.575 | 5.962 | 4.478 | 2.830 | 0.478 |
|  |  | 7 | 2.482 | 8.751 | 7.231 | 2.305 | 1.421 |
|  |  | 8 | 0.870 | 4.339 | 3.878 | 2.381 | 0.388 |
|  |  | 9 | 0.818 | 4.531 | 3.535 | 2.852 | 2.333 |
|  |  | 10 | 0.040 | 0.873 | 0.701 | 0.593 | 0.806 |
|  | 2025 | 5 | 0.332 | 1.500 | 1.100 | 0.919 | 1.274 |
|  |  | 6 | 0.991 | 4.052 | 4.139 | 2.353 | 0.279 |
|  |  | 7 | 1.525 | 4.106 | 4.963 | 4.058 | 0.249 |
|  |  | 8 | 0.850 | 5.108 | 4.256 | 2.813 | 0.080 |
|  |  | 9 | 0.489 | 3.543 | 3.099 | 2.073 | 0.023 |
|  |  | 10 | 0.066 | 2.041 | 1.485 | 1.130 | 0.499 |
| Epailyksensuo | 2024 | 6 |  | 1.612 | 1.509 | 0.787 | 0.453 |
|  |  | 7 |  | 2.066 | 2.654 | 1.481 | 0.153 |
|  |  | 8 |  | 1.081 | 1.421 | 1.172 | 0.576 |
|  |  | 9 |  | 1.049 | 1.482 | 0.690 | 0.996 |
|  |  | 10 |  | 0.264 | 0.441 | 0.169 | 0.212 |
|  | 2025 | 5 |  | 0.416 | 0.865 | 0.502 | 0.742 |
|  |  | 6 |  | 1.108 | 1.296 | 0.752 | 0.296 |
|  |  | 7 |  | 2.198 | 1.804 | 1.167 | 0.361 |
|  |  | 8 |  | 1.350 | 1.389 | 0.895 | 0.859 |
|  |  | 9 |  | 1.171 | 1.277 | 0.524 | 0.251 |
|  |  | 10 |  | 0.530 | 0.665 | 0.230 | 0.267 |
| Marttilansuo | 2024 | 6 |  | 3.636 | 3.624 | 0.906 | 0.346 |
|  |  | 7 |  | 3.332 | 3.201 | 1.365 | 0.838 |
|  |  | 8 |  | 2.647 | 1.713 | 0.966 | 1.057 |
|  |  | 9 |  | 2.291 | 1.512 | 1.176 | 0.989 |
|  |  | 10 |  | 0.703 | 0.625 | 0.337 | 0.232 |
|  | 2025 | 5 |  | 0.634 | 0.451 | 0.396 | 0.238 |
|  |  | 6 |  | 2.076 | 2.287 | 1.079 | 0.283 |
|  |  | 7 |  | 4.174 | 3.011 | 1.568 | 0.437 |
|  |  | 8 |  | 2.51 | 1.656 | 1.201 | 0.481 |
|  |  | 9 |  | 2.952 | 1.577 | 1.146 | 0.549 |
|  |  | 10 |  | 2.000 | 0.678 | 0.700 | 0.234 |

Table S5. Emission factors for g CH_4_-C m^-2^ d^-1^ for each surface type. Factors have been rounded to three decimals.

| Site | Year | Time | Bare peat | Dry vegetation | Wet vegetation | Reed | Water |
| --- | --- | --- | --- | --- | --- | --- | --- |
| Joutsensuo | 2024 | 6 | 0.001 | 0.016 | 0.307 | 0.404 | 0.015 |
|  |  | 7 | 0.002 | 0.191 | 0.320 | 0.267 | 0.011 |
|  |  | 8 | 0.000 | 0.427 | 0.121 | 0.428 | 0.001 |
|  |  | 9 | 0.000 | 0.638 | 0.473 | 0.438 | 0.035 |
|  |  | 10 | 0.000 | 0.023 | 0.080 | 0.089 | 0.003 |
|  | 2025 | 5 | 0.002 | 0.017 | 0.023 | 0.102 | 0.019 |
|  |  | 6 | 0.036 | 0.080 | 0.164 | 0.155 | 0.003 |
|  |  | 7 | 0.005 | 0.038 | 0.115 | 0.338 | 0.009 |
|  |  | 8 | 0.001 | 0.029 | 0.120 | 0.313 | 0.002 |
|  |  | 9 | 0.002 | 0.191 | 0.128 | 0.215 | 0.003 |
|  |  | 10 | 0.001 | 0.072 | 0.113 | 0.070 | 0.002 |
| Epailyksensuo | 2024 | 6 |  | 0.059 | 0.087 | 0.047 | 0.007 |
|  |  | 7 |  | 1.105 | 0.231 | 0.119 | 0.024 |
|  |  | 8 |  | 0.062 | 0.167 | 0.129 | 0.004 |
|  |  | 9 |  | 0.063 | 0.230 | 0.066 | 0.012 |
|  |  | 10 |  | 0.026 | 0.068 | 0.006 | 0.001 |
|  | 2025 | 5 |  | 0.029 | 0.091 | 0.041 | 0.020 |
|  |  | 6 |  | 0.057 | 0.089 | 0.050 | 0.009 |
|  |  | 7 |  | 0.120 | 0.143 | 0.158 | 0.017 |
|  |  | 8 |  | 0.058 | 0.154 | 0.095 | 0.026 |
|  |  | 9 |  | 0.044 | 0.110 | 0.050 | 0.002 |
|  |  | 10 |  | 0.030 | 0.072 | 0.017 | 0.004 |
| Marttilansuo | 2024 | 6 |  | 0.275 | 0.243 | 0.014 | 0.004 |
|  |  | 7 |  | 0.290 | 0.329 | 0.024 | 0.016 |
|  |  | 8 |  | 0.246 | 0.134 | 0.034 | 0.015 |
|  |  | 9 |  | 0.248 | 0.121 | 0.040 | 0.011 |
|  |  | 10 |  | 0.071 | 0.324 | 0.006 | 0.001 |
|  | 2025 | 5 |  | 0.049 | 0.029 | 0.007 | 0.004 |
|  |  | 6 |  | 0.159 | 0.139 | 0.020 | 0.005 |
|  |  | 7 |  | 0.320 | 0.362 | 0.585 | 0.017 |
|  |  | 8 |  | 0.125 | 0.132 | 0.062 | 0.004 |
|  |  | 9 |  | 0.104 | 0.050 | 0.035 | 0.004 |
|  |  | 10 |  | 0.065 | 0.032 | 0.008 | 0.002 |

Table S6. Emission factors for μg N_2_O-N m^-2^ d^-1^ for each surface type. Factors have been rounded to zero decimals.

| Site | Year | Time | Bare peat | Dry vegetation | Wet vegetation | Reed | Water |
| --- | --- | --- | --- | --- | --- | --- | --- |
| Joutsensuo | 2024 | 6 | 18 | -10 | 37 | 101 | 90 |
|  |  | 7 | 50 | 54 | 207 | -47 | -120 |
|  |  | 8 | 14 | -52 | 66 | -99 | -26 |
|  |  | 9 | -31 | -95 | -60 | -152 | -115 |
|  |  | 10 | -4 | -30 | -18 | -7 | 27 |
|  | 2025 | 5 | 1 | -61 | -17 | -1 | -42 |
|  |  | 6 | 8 | -34 | 60 | 1 | -22 |
|  |  | 7 | 69 | -86 | 50 | -25 | -11 |
|  |  | 8 | -2 | 20 | 85 | -88 | 22 |
|  |  | 9 | 45 | -25 | 33 | 8 | 16 |
|  |  | 10 | -37 | 5 | -4 | -10 | -31 |
| Epailyksensuo | 2024 | 6 |  | 22 | -25 | 64 | 10 |
|  |  | 7 |  | -79 | -40 | 20 | -10 |
|  |  | 8 |  | -15 | -46 | -34 | -35 |
|  |  | 9 |  | -94 | -106 | -41 | -25 |
|  |  | 10 |  | -15 | -15 | 12 | 16 |
|  | 2025 | 5 |  | -97 | -67 | 22 | -16 |
|  |  | 6 |  | 17 | -6 | -116 | 10 |
|  |  | 7 |  | 26 | -20 | 60 | 41 |
|  |  | 8 |  | 34 | -38 | -22 | -2 |
|  |  | 9 |  | 11 | -72 | -19 | 27 |
|  |  | 10 |  | 25 | -68 | -6 | -67 |
| Marttilansuo | 2024 | 6 |  | -25 | -20 | 206 | 122 |
|  |  | 7 |  | 51 | -46 | 22 | 13 |
|  |  | 8 |  | -17 | -137 | -23 | -90 |
|  |  | 9 |  | -85 | -99 | -100 | -111 |
|  |  | 10 |  | -60 | -34 | 97 | 57 |
|  | 2025 | 5 |  | -9 | -140 | -11 | 3 |
|  |  | 6 |  | -1 | -52 | 27 | 37 |
|  |  | 7 |  | -11 | -22 | -22 | 80 |
|  |  | 8 |  | 19 | -23 | -24 | 20 |
|  |  | 9 |  | 11 | -43 | 6 | 25 |
|  |  | 10 |  | -42 | -3 | 36 | 7 |

Figure S1. Average plant-functional-type covers (%) among surface types within each site.

Figure S2. Boxplots from all *R*_eco_ measurements for each site and surface type. Stars indicate statistical significance (pairwise Mann-Whitney U test) between different groups, with p-values of ≤ 0.05 (*), ≤ 0.01 (**), ≤ 0.001 (***) and ≤ 0.0001 (****). ns indicates that the p-value is > 0.05. Kruskal-Wallis test indicate that groups differ from each other in all sites statistically significantly (p-value < 0.0001).

Figure S3. Boxplots from all CH_4_-flux measurements for each site and surface type. Stars indicate statistical significance (pairwise Mann-Whitney U test) between different groups, with p-values of ≤ 0.05 (*), ≤ 0.01 (**), ≤ 0.001 (***) and ≤ 0.0001 (****). ns indicates that the p-value is > 0.05. Kruskal-Wallis test indicate that groups differ from each other in all sites statistically significantly (p-value < 0.0001).

Figure S4. Boxplots from all N_2_O-flux measurements for each site and surface type. Stars indicate statistical significance (pairwise Mann-Whitney U test) between different groups, with p-values of ≤ 0.05 (*), ≤ 0.01 (**), ≤ 0.001 (***) and ≤ 0.0001 (****). ns indicates that the p-value is > 0.05. Kruskal-Wallis test indicated that groups differ from each other in all sites statistically significantly (p-value < 0.01).

Figure S5. Spatially cross-validated surface type %-cover predictions and observations for each site. Black line indicates 1:1 line. Bare peat was not classified and modelled in Epäilyksensuo.
